# The Impact of Caregiving on Successful Ageing of Informal Carers: A Qualitative Study among Respiratory Patients’ Caregivers

**DOI:** 10.3390/healthcare11050715

**Published:** 2023-02-28

**Authors:** Snježana Benko Meštrović, Iva Šklempe Kokić, Adriano Friganović, Sabina Krupa, Dijana Babić, Erika Zelko, Dušan Đorđević

**Affiliations:** 1 Special Hospital for Pulmonary Disease, 10000 Zagreb, Croatia; 2 Faculty of Health Studies, University of Rijeka, 51000 Rijeka, Croatia; 3 Faculty of Kinesiology, Josip Juraj Strossmayer University of Osijek, 31000 Osijek, Croatia; 4 Department of Anesthesiology and Intensive Medicine, University Hospital Centre Zagreb, 10000 Zagreb, Croatia; 5 Department of Nursing, University of Applied Sciences, 10000 Zagreb, Croatia; 6 Institute of Health Sciences, College of Medical Sciences, University of Rzeszow, 35-310 Rzeszow, Poland; 7 Magdalena–Clinic for Cardiovascular Disease, 49217 Krapinske Toplice, Croatia; 8 Institute for General Medicine, Johannes Kepler University Linz, Huemerstrasse 5-10, 4020 Linz, Austria; 9 Faculty of Sport and Physical Education, University of Niš, 18000 Niš, Serbia

**Keywords:** informal caregiving, chronic respiratory failure, successful ageing, qualitative study

## Abstract

Providing intensive informal caregiving can cause caregivers’ overburden, possibly impacting successful ageing factors such as physical and mental health and social life. This article aimed to investigate how informal caregivers experience the impact of providing care for chronic respiratory patients on the process of caregiver ageing. A qualitative exploratory study was performed using semi-structured interviews. The sample comprised 15 informal caregivers who provided intensive care for patients with chronic respiratory failure for more than six months. They were recruited while accompanying the patients on their examination for chronic respiratory failure in Special Hospital for Pulmonary Disease in Zagreb between January 2020 and November 2020. Semi-structured interviews were used with informal caregivers, and interview transcripts were analysed by the inductive thematic analysis method. Similar codes were organised into categories, and categories were grouped into themes. Two themes were identified in the domain of physical health relating to informal caregiving activities and inadequate treatment of their difficulties, three themes were in the domain of mental health relating to satisfaction with the recipient of care and feelings, and two themes were in the domain of social life, relating to social isolation and social support. Informal caregivers of patients with chronic respiratory failure experience a negative impact on the factors that contribute to the successful ageing of informal caregivers. The results of our research suggest that caregivers need support in maintaining their own health and social inclusion.

## 1. Introduction

According to the data on informal care collected through the European Quality of Life Survey among close to 37,000 people from 33 countries, it is obvious that a lot of people identify themselves as informal caregivers. For example, as much as 34% of the population in Greece, 30% in Belgium, 10% in Austria, Bulgaria, Ireland, and 15% in Slovenia care for disabled relatives, neighbours, or friends at least once a week, There is no data for Croatia [1,2]. It is important to highlight that there is no consensus in the literature about the definition of informal caregiving, so it could be a limitation in determining the exact number of informal caregivers. The WHO does not provide its official definition of “informal caregiving” but in the context of global cost for informal care, they use the term “unpaid” task [3]. According to the United Nations, “informal caregiving” primarily stands for any non-professional care provided by family members, friends, neighbours, or other persons caring for people with long-term care needs at all ages, usually in private households [4]. OECD considers “informal caregivers” only as family members, friends, or uncompensated volunteers who provide support to care recipients on a regular basis and who do not receive cash or other benefits beyond those intended to support caring activities [5].

Considering the fact that the incidence and prevalence of chronic diseases are increasing and that individuals prefer being cared for at home, the need for informal care is expected to grow in the future [6]. This is also supported by recorded increase in the number of family caregivers in the United States of 9.5 million from 2015 to 2020, which means that nearly one in five Americans (19%) are providing unpaid care to an adult with health or functional need [7]. On the other hand, it is essential to note that due to providing care, informal caregivers usually do not have enough time to take care of their own health or to be socially active, which can have a negative impact on informal caregivers’ physical and psychological wellbeing, leading to their overburden [8]. It is well known that because of the increasing lifespan, public health and health care systems promote preservation of functional capacity for as long as possible, emphasising independent functioning and optimal physical and mental health [9]. Those efforts have resulted in the concept of successful ageing that appeared in the literature in the early 1960s [10]. Successful ageing means reaching the maximum personal level of physical, mental, and social wellbeing in old age with three components met: disease avoidance, social inclusion, and maintaining a high level of cognitive and bodily functions [11,12]. Family caregivers are in an ideal position to provide insights into the meaning of successful ageing since their everyday lives are profoundly affected by caring for family members who are unwell and/or disabled. If we know that informal caregivers are mostly females aged 45–65 [13,14], it is questionable whether they have a chance for successful ageing.

Lack of family and social support, and insufficient coping skills for everyday challenges of care, are among the most important predictors of informal caregiver burden. Overburdened informal caregivers are at a high risk of developing health impairments. This means that, if they disregard their own health to focus on the health and support of the person they are caring for, caregivers’ health may worsen to the point that they are no longer able to perform the caring duty [15,16,17]. Other important predictors of informal caregiver burden are the physical and mental conditions of patients. Chronic respiratory failure patients, as well as their informal caregivers, are confronted with many symptoms such as dyspnoea and fatigue and multiple limitations in activities of daily living [18]. Respiratory failure is a clinical condition that occurs when the respiratory system fails to maintain its main function and usually requires providing support with oxygenation and ventilation [19].

Caregivers of patients with severe symptoms and more functional disabilities, especially during exacerbation of the disease, perceived that they had poorer physical and mental health-related quality of life [6,20]. Hence, the aim of our research was to investigate how informal caregivers of chronic respiratory failure patients experienced providing care and how the caring role impacted successful ageing: physical and mental health, and social life. With this research, we aimed to fill the literature gap and to contribute with new knowledge in the field of caregiving for chronic respiratory patients. No studies were found that specifically addressed how chronic respiratory failure patients’ caregivers experienced the impact of caregiving to their ageing.

## 2. Materials and Methods

### 2.1. Study Subjects

The study was conducted from January to November 2020. The inclusion criteria were providers of informal care for people with chronic respiratory failure, who used some kind of respiratory support for more than 6 months (20+ h/week), self-identification as primary informal caregivers, with no financial compensation for care (except parents who receive 500 euros per month). The exclusion criteria were the provision of informal care to a person who has another unrelated illness that requires care, and receiving compensation/salary for providing care. During patient follow-up examinations for chronic respiratory failure in the Special Hospital for Pulmonary Disease, informal caregivers who met the criteria were asked to participate in this qualitative study.

Informal care included assistance for respiratory patients in activities of daily living (cooking, shopping, bathing, dressing, moving from bed to bathroom, and feeding) and conducting medical interventions (giving oral and parenteral drug therapy; assistance in using walking aids; preparing special food; caring for wounds; use of equipment to measure glucose, oxygen, temperature, and blood pressure; incontinence-related activities; aspiration of bronchial secretions; tube or percutaneous gastrostomy feeding; and control of devices such as ventilators and/or oxygen concentrators). Ethics approval was granted by the Special Hospital for Pulmonary Disease Ethics Committee (No. 3000/2019). Participants provided written informed consent. Likewise, researchers followed all recommendations of the Helsinki Declaration.

### 2.2. Methods

We conducted 15 semi-structured interviews with informal caregivers. Data saturation was achieved by the 15th interview. The interview questions were developed after literature research and with suggestions from the focus group of experts relating to the research topic (Appendix A). The focus group, comprising a gerontologist, psychologist, and physiotherapist, defined key factors important for successful ageing. Considering that physical/mental health and social life are crucial domains related to successful ageing, the semi-structured interview consisted of 15 main questions with sub-questions, five of them related to physical health, five related to mental health, and five related to social life. If informal caregivers agreed to participate in the research, they self-completed a sociodemographic questionnaire and orally responded to the semi-structured interview to provide greater and deeper insight into their experiences. The questions included their experience of how providing care influenced their physical and mental health and social life. Interviews were taken in the Special Hospital for Pulmonary Disease in Zagreb during a regular medical check-up of the care recipient or at the caregiver home and were carried out by the chief investigator. Interviews were recorded and not conducted in front of the care recipients. Only the person conducting the interviews (researcher) was aware of the participants’ identities. The same researcher was the one who made transcripts, which did not contain the name or any other identifier but were rather coded as IDI 1, IDI 2, etc. Analysis and coding were done only based on coded transcripts.

### 2.3. Analysis

Every participant had a personal code to ensure absolute anonymity and to follow all ethical considerations. The further process of data analysis excluded participants’ personal data. We digitally taped the interviews and transcribed the recordings verbatim, assigning each interview a unique identifier. We completed an inductive thematic analysis of the transcripts by three researchers (triangulation), beginning after the first interview and using an open coding process [21]. The goal of these interviews and the inductive thematic analysis of their contents was to answer the research questions. In open coding, researchers independently identified codes, then grouped the codes into categories and categories into themes. The open coding process gave us the possibility to identify specific concepts and themes for categorisation and the initial broad thematic domains for data assemblage [21]. They repeated this process for each transcript. All three researchers coded independently, and inconsistencies were resolved by the main researcher. Independent analysis procedures were synthesised at the general meeting, and then, consensus was achieved on the final themes arising from the three different data interpretations. Inconsistencies were resolved by the main researcher.

## 3. Results

The study sample comprised 15 informal caregivers of chronic respiratory failure patients. Eight of the patients used long-term oxygen therapy, four used non-invasive mechanical ventilation and oxygen therapy, and three used invasive mechanical ventilation as respiratory support. The average age of the informal caregivers was 57 (57.11 ± 11.63 years), and the other sociodemographic characteristics of the participants are presented in Table 1.

**Figure 1:**
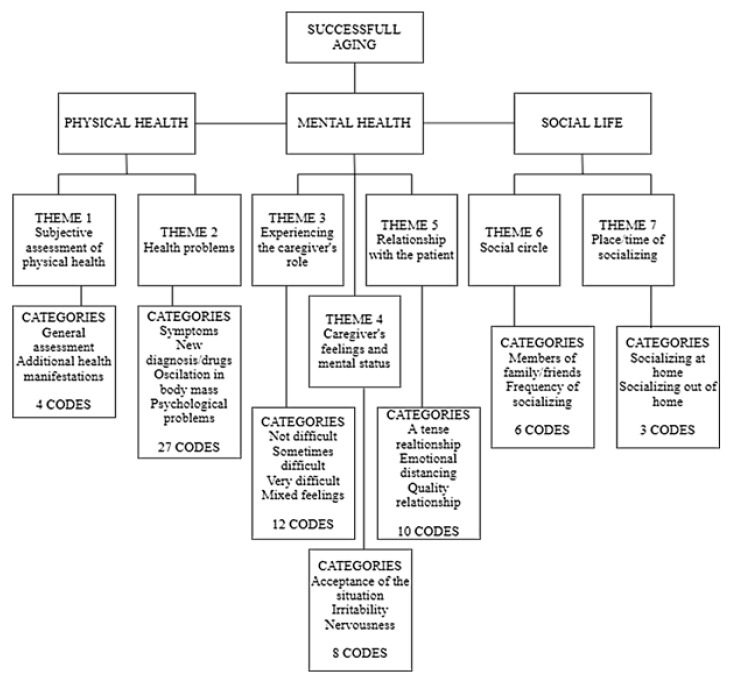
Results of the inductive analysis.

The average duration of the semi-structured interviews was 28 min. An inductive thematic analysis of the interview data resulted in 7 themes, 20 categories, and 70 codes (Figure 1).

In the first theme (subjective assessment of physical health), caregivers assessed their own physical health and noted that they usually were not satisfied with their health conditions. They also reported a lack of free time for physical activity and improper diet as problems. *“Apart from not moving enough, I also eat more. Especially in the last year, I have felt pain in my back, shoulders, and arms more often. (Name withheld) weighs about 13 kg, and it is usually in my arms because it makes it easier to relax.” (Caregiver 1).*

In the second theme (health problems), caregivers reported that, besides not having enough time to engage in physical activity, they also lacked time for their own medical examinations and treatments. They also reported changes in their health status since they took on the role of caregivers, such as discovering new diagnoses, musculoskeletal pain, and weight oscillations. *“I’m sure that I’m not well. But I can’t visit my physician. Not even a gynecologist. Imagine I have no one to leave a sick child to. The only doctors I see are hers. So I really don’t know if I have a disease or not. But my whole body often hurts” (Caregiver 2).*

Participants noted, in particular, problems with gastritis, breathing difficulties, headache, sleep disorders, and fatigue, and it is well known that those physical problems are related to psychological problems. “*I associate some of these changes, such as pain in the head and neck, with a change in lifestyle, which used to be much more active. And there is also tension. Yes, I feel that tension in my head and neck*” *(Caregiver 3).*

In the third theme (experiencing the role of a caregiver), they described how they coped with the role of a caregiver. Sometimes, they feel a heavy burden due to the role of being an informal caregiver and feel that they cannot do it anymore, but because of compassion and a sense of duty, they continue to take care of a sick family member. *“There are days when I think I will not be able to continue. That I will tell my husband and the children that I can’t do it anymore. I’m telling you, I’m tired. And then I look at him and I feel sorry for him. I’m so sorry for him. I don’t know who would take better care of him than I do...“ (Caregiver 4).*

In the fourth theme (caregivers’ feelings and mental status), participants described the feelings the caregiver role evoked in them. Some of them were used to the situation, but most of them felt nervous and worried about the future. *“I think I’m constantly tense. I don’t know what it’s like not to be tense! I’m definitely tenser than before. On the one hand, I am more nervous because I know that (name withheld) depends on me the most and I often don’t have much time for myself, no time to sleep, let alone anything else… It’s just that now I’m more afraid for her than a year ago because she had several ugly seizures with a big drop in saturation.“ (Caregiver 5).*

In the fifth theme (relationship with the patient), they described their relationship with the recipient of care. The quality of their relationships usually depended on the state of the relationship before the illness. The mothers of ill children were very patient and sensitive. Spouses who found their marriage harmonious tolerated the role of informal caregivers better than others. *“It used to bother me more, and I resented him more when he was healthy. Now, he is helpless and nailed to the house. He can be demanding at times and wants to be the center of attention all the time, but he has been like that before. What used to bother me doesn’t bother me anymore because the disease calmed him down.” (Caregiver 6).*

*“He can’t do anything anymore. Not even talk. We get along as a patient and a nurse (laughs). It’s the only thing that connects us. My constant concern for him and his need for my help. We agree only on that”.*

*(Caregiver 7)*

*“As in any marriage, we sometimes argue. But I’m gentler in an argument than before my illness (laughs). We actually get along well. The disease did not manage to destroy us”.*

*(Caregiver 8)*

*“Our relationship is quite turbulent. Mom always has to be right and always uses the sentence: I’m seriously ill! And that’s how she always provokes a feeling of remorse in me. But I have to take care of her”.*

*(Caregiver 9)*

In the sixth theme (social circle), participants reported that they did not have social lives, except occasional socialising with family members or medical staff. Only employed caregivers sometimes had coffee with their colleagues. *“The son is in Germany, the daughter sometimes comes. Otherwise, I am all alone. Only people from the ambulance and the hospital.” (Caregiver 6).*

In the seventh theme (Place/time of socialising), participants reported they were usually at home, and the only socialising they experienced was when somebody visited them, which was very rarely, and during the pandemic, it was almost non-existent. *“No one is coming to us in this pandemic anymore. Children and grandchildren say it’s because of the pandemic. But I think that’s an excuse for them. It’s hard for them to watch Dad so helpless. I understand that.” (Caregiver 7).*

## 4. Discussion

Our study aimed to investigate how informal caregivers of chronic respiratory failure patients experience providing care and how the caring role impacted successful ageing: physical and mental health and social life.

According to the results of our research, informal caregivers experience problems with their physical and mental health. Usually, they suffer from musculoskeletal pain, sleep disorders, and weight oscillations. Some of them have been diagnosed with new health conditions as a result of caring for an ill family member. Our results, especially those obtained from caregivers 1, 2, and 3, are likely in line with other authors’ results [22,23,24], who have also obtained that informal caregiver overburden causes immune system weakening, high blood glucose levels, high blood pressure, and sleeping disorders. This could potentially be linked with providing caring interventions, feeling overburdened, lack of free time for their own physical activity, or lack of regular medical examinations and treatments. Likewise, our results correlate with previously published studies about caregiving for a person with advanced illness suffering from breathlessness where informal caregivers also experienced a lack of time for their own needs and self-care [15,25]. Despite the fact that women are less career-oriented and place more considerable importance on non-market activities such as family responsibilities, women who provide ongoing intense care may find it more difficult to focus on the positive aspects of caring [26]. They may feel seriously impaired if they become inactive as a result of their caring responsibilities [27]. In that regard, caregivers encounter all the problems of organising a care program with minimal or no support. As a result, they are burdened with far more responsibility, resulting in an excessive amount of emotional pressure [28].

Caring interventions such as transfers and bathing lead to physical overburden, which includes headaches, musculoskeletal pain, obesity, and high levels of stress hormones [29]. These diverse challenges could be the main cause of caregiver mental risk. The results of other research [22,24,30,31,32] indicated that informal caregivers are at a high risk of developing mental disorders, such as depression and anxiety, especially if the caregiver and patient are relatives [33]. In our case, participants often felt a heavy burden due to the role of the informal caregiver, and there were caregivers who were relatives, which is in line with other research results. Likewise, several authors [34,35,36,37] agreed on the fact that intensive care, feelings of being overburden, lack of social and emotional support, being female, and emotionally focused confrontation with caregiving problems are predictors for both depression and anxiety. Zarit and Reamy [38] believed that stress and burden may lead to burnout, which will further impair the caregiver’s mental state. Another fact that must be considered is that women are more exposed than men to the stress generated by informal caregiving, with men reacting differently to stressful situations than women [39].

Our participants also experienced social isolation because of a lack of free time for social lives. Sometimes, care recipients can feel uneasy when signs of poor health become visible in public. If he/she does not want to be seen in such a condition, the caregiver cannot go outside, and visiting friends are not welcome [25]. Considering that informal caregivers’ satisfaction with their social lives may prevent the negative impacts of informal caregiving, other opportunities for their social inclusion should be considered. This could also include virtual technology [22,40,41,42].

In addition to the previous, since our participants’ average age was 57 (57.11 ± 11.63 years) and comprised mainly women, we could say that there is a link in terms of age and gender with European informal care [1], as well as with the OECD report [2]. However, further research is needed to complete the gaps and update the insufficient data in this part of Europe.

The conducted study had some limitations. Participants were recruited only in one hospital, and they all lived in the capital of Croatia (Zagreb), which could mean that our sample was not representative of all caregiver populations in the country. Furthermore, the majority of our sample consisted of women. The types of caregiver recipient’s respiratory support were different (long-term oxygen therapy, non-invasive mechanical ventilation and oxygen therapy, and invasive mechanical ventilation), so it could possibly be expected that the caregiver’s experience is not the same if they take care of somebody who uses long-term oxygen therapy or invasive mechanical ventilation. Furthermore, there was a conceptual framework deficiency that guided the interview questions, which further benefited the data processing.

## 5. Conclusions

Informal caregivers experience negative impacts on the factors that contribute to the successful ageing of informal caregivers of patients with chronic respiratory failure: physical and mental health and social life. Due to the demanding nature of providing intensive care, they usually do not have enough time to take care of their own health or have social lives. Caregivers’ needs for maintaining their own health are not being met effectively; they require aid and emotional support to prevent care recipient institutionalisation. In that regard, it is essential to identify informal caregivers whose physical/mental health and social life are affected by providing care and provide them with appropriate support in line with their needs.

Based on the fact that we conducted a qualitative study, future ones should focus on a longitudinal approach, mainly because we did not explore the impact that arose from the caregiving role (e.g., caregiver’s resilience, patience, tolerance, inner strength, improving relationship with person that receiving care, etc.).

## Figures and Tables

**Table 1:** Sociodemographic characteristics of the participants.

Variables		N
Gender	Male	1
	Female	14
Work Status	Full-time job	3
	Parent caregiver	2
	Retirement	8
	Unemployed	2
Relationship with the patient	Daughter	3
	Son	1
	Mother	2
	Wife	9

## Data Availability

Not applicable.

## Appendix A. Semi-Structured Interview Questions

1. Please describe your typical day.
2. Do you have trouble sleeping? If so, what causes the difficulties? Can you remember how you slept before the onset of the disease in the family?
3. Do you practice any physical activity? If so, what kind of physical activity and how often in the past month?
4. Can you remember some activity you used to practice and now you are not able to? If you no longer perform certain physical activities, what are the reasons for that?
5. How do you assess your physical health? Has there been any change since you took on the role of caregiver? Do you go to regular medical examinations?
6. How do you feel about your role as a caregiver? Do you find this role difficult? If this role burdens you, what do you consider to be the main cause of that burden?
7. What kind of relationship do you have with the care recipient? Is there anything in that relationship that has changed since you became a care provider? Do you agree with him/her? If something particularly bothers you in that relationship, what is it?
8. Does the role of the caregiver and the activities related to that role affect how you feel? Are you more nervous than before?
9. Are you bothered by anything related to providing care? Do you talk to anyone about any difficulties you may face? Do you unburden yourself to anyone?
10. Have you ever sought or considered seeking professional, psychological help? If so, what motivated you to seek help?
11. Have you been hanging out with relatives or friends lately? Where do you hang out most often?
12. Have you been traveling lately (going on vacation or visiting relatives)? Has there been any change since you took on the role of caregiver? What would help you travel somewhere?
13. How would your family members describe you? Have you changed since you took on the role of caregiver?
14. Do family members or friends help you with caring for a sick family member? Do you need additional help?
15. Have you had time lately to engage in some free activities (hobbies, visiting exhibitions, theater...)? If no, what was the reason for that?
